# Une cause inhabituelle d’ictère

**DOI:** 10.11604/pamj.2018.31.72.16967

**Published:** 2018-10-02

**Authors:** Salamata Diallo, Boundia Djiba, Marie Louise Bassène, Mamadou Ngoné Gueye, Mame Aissé Thioubou, Mariéme Poléle Fall, Cheikh Ahmadou Bamba Cissé, Daouda Dia, Mouhamadou Mbengue, Mamadou Lamine Diouf

**Affiliations:** 1 Service d’Hépato-Gastroentérologie de l’Hôpital Aritide Le Dantec, Dakar, Sénégal; 2 Service de Médecine Interne de l’Hôpital Aritide Le Dantec, Dakar, Sénégal; 3 Service d’Hépato-Gastroentérologie de l’Hôpital Général de Grand Yoff, Dakar, Sénégal

**Keywords:** Hyperthyroïdie, Ictère, Sénégal, Hyperthyroidism, jaundice, Senegal

## Abstract

L'atteinte hépatique est fréquente au cours de l'hyperthyroïdie. Elle est le plus souvent asymptomatique. Une hyperthyroïdie révélée par un ictère est rarement décrite dans littérature. Nous vous rapportons une observation à Dakar (Sénégal). Il s'agissait d'un patient de 52 ans qui avait consulté notre service pour un ictère associé à un prurit. Les explorations biologiques montraient une augmentation des alanine aminotranférases (1,1 N), des aspartate aminotranférases (1,5 N), des phosphatases alcalines (3 N), des gamma glutamyl transférases (1,3 N) et de la bilirubinémie (22 N). L'échographie abdominale était normale. Une cause toxique ou médicamenteuse, un obstacle sur les voies biliaires, une hépatite virale ou auto immune ainsi qu'une cholangite biliaire primitive ont été exclu. Le dosage des hormones thyroïdiennes montrait une élévation de la T4 libre à 24 ng/dL (9-20 ng/dL) et un taux plasmatique indétectable de TSH inférieure à 0,01μUI/mL (0,35-4,94 UI/mL). Les anticorps anti récepteurs de la TSH étaient positifs à 7,04 UI/L (N < 1,75 UI/L). L'échographie thyroïdienne objectivait un goitre diffus homogène hypervasculaire. Le diagnostic d'une atteinte hépatique secondaire à une maladie de Basedow sans dysfonction cardiaque était retenu. L'évolution clinique et biologique était favorable sous carbimazole. L'ictère peut être révélatrice d'une hyperthyroïdie. La recherche des signes cliniques et biologiques d'une hyperthyroïdie est obligatoire devant un ictère inexpliqué.

## Abstract (en)

Hepatic impairment is common during hyperthyroidism. It is most often asymptomatic. Hyperthyroidism revealed by jaundice has been rarely described in the literature. We here report the case of a 52-year old patient in Dakar (Senegal) presenting with jaundice associated with pruritus. Laboratory tests showed elevated alanine aminotransferases (1.1 N), aspartate aminotransferase(1.5 N), alkaline phosphatases (3 N), gamma glutamyl transferases (1.3 N) and bilirubinemia (22 N). Abdominal ultrasound was normal. A toxic or drug-related cause, bile duct obstruction, viral or autoimmune hepatitis as well as primary biliary cholangitis were excluded. The dosage of thyroid hormones showed elevated free T4, 24 ng/dL (9-20 ng/dL) and undetectable plasma TSH less than 0.01μUI/mL (0,35-4,94 IU/mL). TSH receptor antibodies were positive 7.04 IU/L (n < 1.75 IU/L). Thyroid ultrasound objectified diffuse homogeneous hypervascular goiter. The diagnosis of hepatic impairment secondary to Graves-Basedow disease without cardiac dysfunction was retained. Clinical outcome and laboratory test results were favorable under carbimazole. Jaundice can be an indicator of hyperthyroidism. An investivation of clinical signs and laboratory parameters for hyperthyroidism is essential in patients with unexplained jaundice.

## Introduction

L'atteinte hépatique est fréquente au cours de l'hyperthyroïdie. Elle est le plus souvent asymptomatique. Les manifestations cliniques notamment l'ictère sont rares. Cette atteinte hépatique peut être due à une crise aigue thyréotoxique, à une hyperthyroïdie avec une atteinte cardiaque, à une association avec une pathologie hépatique ou à la toxicité des antithyroïdiens de synthèse. Rarement, elle est en rapport avec une thyrotoxicose isolée. Nous rapportons une observation d'hyperthyroïdie sans atteinte cardiaque révélée par un ictère.

## Patient et observation

M. M.A.B. âgé de 52 ans a consulté en Juin 2017 notre service pour un ictère cutanéo-muqueux évoluant depuis environ 1 mois associé à un prurit généralisé et à une perte de poids de 3 kg. L'interrogatoire ne retrouvait aucun antécédent particulier, pas de notion d'hépatopathie ni de prise médicamenteuse. Le patient était non éthylique et non tabagique. L'examen clinique objectivait un ictère conjonctival franc sans lésions de grattage. La fréquence cardiaque était de 104 battements par minute. Le reste de l'examen clinique était normal. Il n'y avait pas de goitre, pas d'exophtalmie, ni d'hépatosplénomégalie, ni de signes d'insuffisance cardiaque. Les explorations fonctionnelles hépatiques révélaient une cholestase avec des phosphatases alcalines à 3N, des gamma glutamyl transférase à 1,3 N, une bilirubinémie 22 N à prédominance conjuguée (80%) et une cytolyse (ASAT à 1,5 N, ALAT à 1,1 N). Le TP était à 100 % et l'albuminémie à 41 g/ l. Les sérologies virales B, C étaient négatives. La recherche des anticorps anti mitochondries, anti muscles lisses, anti LKM1 et anti nucléaires étaient négatives. L'échographie abdominale était normale. La Bili-IRM montrait une absence de dilatation des voies biliaires intra et extra hépatique ; le pancréas et le foie étaient normaux. La ponction biopsie hépatique révélait une cholestase prédominante au niveau centro lobulaire sans infiltrat inflammatoire associé à une petite fibrose sinusoïdale ; il n'y avait pas de granulome ou de stéatose. Le dosage des hormones thyroïdiennes montrait une élévation de la T4 libre à 24 ng/dL (9-20 ng/dL) et un taux plasmatique indétectable de TSH inférieure à 0,01μUI/mL (0,35-4,94 UI/ml). Les anticorps anti récepteurs de la TSH étaient positifs 7,04 UI/L (N < 1,75UI/L). L'échographie thyroïdienne objectivait un goitre diffus homogène hypervasculaire. Un traitement par carbimazole et propanolol fut instauré. L'évolution clinique était marquée par la régression de l'ictère, du prurit et une prise de poids de 5 kg. Les explorations biologiques réalisées 3 mois après le début du traitement montraient une normalisation de la T4 libre, de la TSH, des transaminases, de la bilirubine, des GGT et une baisse du taux des phosphatases alcalines à 1,3 N.

## Discussion

L'atteinte hépatique au cours de l'hyperthyroïdie est fréquente. Les interactions entre le foie et la thyroïde sont nombreuses. Le foie joue un rôle important dans le métabolisme des hormones thyroïdiennes et celles-ci modulent au niveau hépatique la croissance et la différenciation cellulaire ainsi que le métabolisme de la bilirubine et des acides biliaires. Selon la littérature, l'atteinte hépatique est présente dans 37 à 78 % des cas [1, 2]. Elle est plus souvent asymptomatique. En l'absence de décompensation cardiaque l'ictère est objectivé dans environ 5 à 11 % des cas [3, 4]. Sur le plan biologique, l´anomalie la plus fréquente est une élévation de l´activité sérique des phosphatases alcalines, observée dans 64 % à 70 % des cas [5, 6]. Elle est généralement secondaire à une élévation de la fraction osseuse de l'enzyme [6]. Les GGT sont élevées dans 17 % des cas [6] et les ALAT dans 28 à 37 % des cas [6, 7]. L'atteinte hépatique peut être due à une crise aigue thyréotoxique, à une hyperthyroïdie avec une décompensation cardiaque, à une association avec une pathologie hépatique ou à la toxicité des antithyroïdiens de synthèse. Notre patient présente une maladie de Basedow compliquée d'une atteinte hépatique sans dysfonction cardiaque. L'atteinte hépatique était rattachée à la thyrotoxicose car une cause toxique ou médicamenteuse, un obstacle au niveau des voies biliaires, une hépatite virale B ou C, une hépatite auto immune ou une cholangite biliaire primitive ont été exclus. Il n'y avait pas de signes en faveur d'une crise aigue thyréotoxique. L'évolution favorable après traitement par anti thyroïdiens de synthèse était également un argument en faveur de cette atteinte hépatique au cours de la thyréotoxicose. Une atteinte hépatique secondaire à une thyréotoxicose isolée sans atteinte cardiaque a été décrite pour la première fois en 1874 [8]. Depuis lors plusieurs cas ont été décrits [9-11]. Les mécanismes responsables de cette atteinte sont mal connus. Plusieurs hypothèses ont été émises.Un effet toxique direct des hormones thyroïdiennes n´a jamais été démontré [12]. L´état hyper métabolique caractéristique de l´hyperthyroïdie pourrait augmenter la demande hépatique en oxygène [13]. L'augmentation de la consommation d´oxygène hépatique sans augmentation parallèle du débit sanguin hépatique entraine une hypoxie tissulaire qui pourrait expliquer les anomalies biologiques et la cholestase [14]. Chez ces patients, la biopsie hépatique montre des anomalies non spécifiques avec une cholestase intrahépatocytaire surtout centrolobulaire, un infiltrat lymphocytaire des espaces portes, une vacuolisation, une hyperplasie des cellules de Kupffer , une fibrose autour des veines sus hépatiques [4, 15].

## Conclusion

La révélation d'une hyperthyroïdie par un ictère est rare. Notre observation illustre l'importance de rechercher des signes cliniques et biologiques d'hyperthyroïdie devant tout ictère inexpliqué.

## Conflits d’intérêts

Les auteurs ne déclarent aucun conflit d'intérêts.

## References

[1] Gürlek A, Çobankara V, Bayraktar M (1997). Liver tests in hyperthyroidism: effect of antithyroid therapy. J Clin Gastroenterol..

[2] He K, Hu Y, Xu XH, Mao XM (2014). Hepatic dysfunction related to thyrotropin receptor antibody in patients with Graves' disease. Exp Clin Endocrinol Diabetes..

[3] Bayraktar M, Van DT (1997). Abnormalities in measures of liver function and injury in thyroid disorders. Hepatogastroenterology..

[4] Fong TL, McHutchison JG, Reynolds TB (1992). Hyperthyroidism and hepatic dysfunction: a case series analysis. J Clin Gastroenterol..

[5] Cooper DS, Kaplan MM, Ridgway EC, Maloof F, Daniels GH (1979). Alkaline phosphatase isoenzyme patterns in hyperthyroidism. Ann Intern Med..

[6] Huang M-J, Li K-L, Wei JS, Wu S-S, Fan K-D, Liaw Y-F (1994). Sequential liver and bone biochemical changes in hyperthyroidism: prospective controlled follow-up study. Am J Gastroenterol..

[7] Thompson P, Strum D, Boehm T, Wartofsky L (1978). Abnormalities of liver function tests in thyrotoxicosis. Mil Med..

[8] Habershon SO (1874). Exophthalmic goiter, heart disease, jaundice, death. Lancet..

[9] Usta Y, Massaad J, Parekh S, Knecht K (2013). Severe cholestatic jaundice secondary to hyperthyroidism. Int J Case Rep Images IJCRI..

[10] Soysal D, Tatar E, Solmaz S, Kabayeğit O, Tunakan M, Unsal B (2008). A case of severe cholestatic jaundice associated with Graves' disease. Turk J Gastroenterol..

[11] Akande TO, Balogun WO (2013). A report of three cases of jaundice with thyrotoxicosis. Afr Health Sci..

[12] Yao JD, Gross JB, Ludwig J, Purnell DC (1989). Cholestatic jaundice in hyperthyroidism. Am J Med..

[13] Goglia F, Liverini G, Lanni A, Barletta A (1988). Mitochondrial DNA, RNA and protein synthesis in normal, hypothyroid and mildly hyperthyroid rat liver during cold exposure. Mol Cell Endocrinol..

[14] Fagiuoli SR, Van Thiel DH (1993). The liver in endocrine disorders. Liver Syst Dis..

[15] Sola J, Pardo-Mindan FJ, Zozaya J, Quiroga J, Sangro B, Prieto J (1991). Liver changes in patients with hyperthyroidism. Liver..

